# Two states of magnetotail dipolarization fronts: A statistical study

**DOI:** 10.1002/2014JA020380

**Published:** 2015-02-16

**Authors:** D Schmid, R Nakamura, F Plaschke, M Volwerk, W Baumjohann

**Affiliations:** 1 Space Research Institute, Austrian Academy of SciencesGraz, Austria; 2 University of Graz, NAWI GrazGraz, Austria

**Keywords:** dipolarization front

## Abstract

We study the ion density and temperature in the predipolarization and postdipolarization plasma sheets in the Earth's magnetotail using 9 years (2001–2009) of Cluster data. For our study we selected cases when Cluster observed dipolarization fronts (DFs) with an earthward plasma flow greater than 150km/s. We perform a statistical study of the temperature and density variations during the DF crossings. Earlier studies concluded that on average, the temperature increases while the density decreases across the DF. Our statistical results show a more diverse picture: While ∼54% of the DFs follow this pattern (category A), for ∼28% the temperature decreases while the density increases across the DF (category B). We found an overall decrease in thermal pressure for category A DFs with a more pronounced decrease at the DF duskside, while DFs of category B showed no clear pattern in the pressure change. Both categories are associated with earthward plasma flows but with some difference: (1) category A flows are faster than category B flows, (2) the observations indicate that category B flows are directed perpendicular to the current in the near-Earth current sheet while category A flows are tilted slightly duskward from this direction, and (3) the background *B*_*z*_ of category B is higher than that of category A. Based on these results, we hypothesize that after reconnection takes place, a bursty bulk flow emerges with category A characteristics, and as it travels earthward, it further evolves into category B characteristics, which is in a more dipolarized region with slower plasma flow (closer to the flow-braking region).

## 1. Introduction

The magnetosphere's tail is characterized by the two large lobes of nearly parallel but oppositely directed magnetic field lines. The two lobes are separated by the high-density plasma sheet/current sheet. Within the plasma sheet (and neutral sheet), transient increases in the magnetic field component perpendicular to the sheet (i.e., *B*_*z*_ in geocentric solar magnetospheric coordinates (GSM)) are a common feature. They have been observed for decades and are typically called magnetotail dipolarizations or dipolarization fronts (terms used as synonyms in this paper). They are usually associated with plasma flows in either earthward or tailward direction and may be associated with substorms [e.g., *Baumjohann et al.*, 1999; *Sergeev et al.*, 2012a] and play an important role in the flux transport in the tail [e.g., *Baumjohann*, [Bibr b3],[Bibr b4]; *Volwerk et al.*, 2008].

Their origin is thought to be reconnection of stretched magnetic field lines. The newly connected field lines will move toward Earth, releasing magnetic tension. This creates fast earthward flows called bursty bulk flow (BBF) [e.g., *Angelopoulos et al.*, 1992; *Baumjohann et al.*, 2002] and a sharp increase in the magnetic field component perpendicular to the current sheet in the tail (often associated with a strong *B*_*z*_ component), making it look like a more dipole-like field, hence the name dipolarization front [e.g., *Nakamura et al.*, [Bibr b17],[Bibr b18]]. Typically, the increase in *B*_*z*_ is preceded by a decrease in *B*_*z*_, sometimes even turning negative [*Ohtani et al.*, 2004]. These asymmetric bipolar *B*_*z*_ variations have been observed in a large range of the magnetotail (−30*R*_*E*_ ≤ *X* ≤ −5*R*_*E*_) [see, e.g., *Ohtani et al.*, 2004].

A possible interpretation of DFs is that they are thin boundaries between an earthward propagating plasma bubble (a flux tube with reduced flux tube entropy) and the ambient plasma sheet [e.g., *Pontius and Wolf*, 1990]. These bubbles propagate earthward until the specific entropies inside and outside the flux tube are equal [e.g., *Sergeev et al.*, 2012b]. This bubble model is supported by results of *Kim et al.* [2010], who preformed a statistical study of the properties of flow bursts (FB) in the magnetotail. Most of their studied FBs were associated with depletion of ion density and flux tube entropy relative to the surrounding medium. They also found that larger density depletion relative to the background is associated with a faster plasma flow. Furthermore, they showed that the temperatures for most of their studied FBs are higher than their surrounding medium temperatures and that the ion pressure decreases relative to the surrounding medium. However, in order to retain pressure balance between the plasma bubble and the surrounding medium, a flux tube with low plasma pressure requires a stronger magnetic field inside [see, e.g., *Li et al.*, 2011]. *Liu et al.* [2013a] called the plasma bubble's strong magnetic field region, which is led by the DF, the dipolarizing flux bundle (DFB). Here we use their nomenclature to describe the region of enhanced magnetic field after the DF.

*Runov et al.* [2011] presented a superposed epoch analysis of DFs. Common features from this study are the following: (1) the northward component of the magnetic field (*B*_*z*_) shows an asymmetric bipolar variation (defining feature), (2) after the DF passage, the ion density decreases to 0.5 of the initial value, (3) the ion temperature increases behind the front, and (4) the ion pressure decreases after the DF, since the temperature increase does not compensate the density decrease. These results are consistent with recent studies by *Liu et al.* [2013b], who also found that the thermal pressure in the equatorial plane is strongest immediately ahead of the DF's leading point and decreases with distance from that point. *Zhou et al.* [2014] simulated the spatial distribution of plasma pressure earthward of a convex DF. The result of this study showed a clear dawn-dusk asymmetry in the pressure, with greater enhancements in front of the DF duskside.

*Schmid et al.* [2011] showed that DFs can be divided into two groups, according to the angle between the plasma flow direction and the magnetic field motion direction, with the angle either smaller or larger than 90°. There is the expected category, in which the plasma and the magnetic field move in the same direction and the dipolarization that is observed comes from the relaxation of the magnetic tension in the stretched field lines as they move earthward. Indeed, about two thirds of their studied events fall into this category. The other category is when the plasma flow is earthward, but the dipolarization motion is tailward. This means that there is a pileup of the magnetic field in the tail.

In this paper, we revisit the properties of DFs, using a recently compiled list of DFs within the FP 7 European Cluster Assimilation Technology (ECLAT) project (http://www.eclat-project.eu). To find the DF events between July and October for the years 2001–2009, similar selection criteria as in *Schmid et al.* [2011] are used. Interestingly, quite a significant amount of DFs within that list are inconsistent with the paradigmatic features of density decrease and temperature increase across the front, although the event search criteria are similar to previous publications [e.g., *Ohtani et al.*, 2004; *Sigsbee et al.*, 2005]. In this study we seek to characterize these “atypical” DFs, compare them to the “typical” ones, and address the question of why they are different and how/where do they originate.

## 2. Data and Event Selection

Our starting point is the DF list compiled within the ECLAT project (see Appendix A), which consists of 1072 events. Each event marks one DF, but each event may have been observed by more than one of the four Cluster spacecraft. Hence, the 1072 events correspond to 1907 single-spacecraft observations (SSOs) by Cluster 1, 3, and 4. Cluster 2 observations are not useful for DF characterization as the plasma instruments on that spacecraft are not working. Plasma moments for Cluster 1 and 3 are provided by the Hot Ion Analyzer (HIA) [*Rème et al.*, 2001] significantly degraded after 2005), whereas for Cluster 4, moments are based on the Ion Composition and Distribution Function Analyser (CODIF) [*Rème et al.*, 2001] (HIA was not operating). Magentic field data obtained by the Flux Gate Magnetometer (FGM) [*Balogh et al.*, 2001] are available for all spacecraft. Throughout the paper, the data are presented in geocentric solar magnetospheric (GSM) coordinate system unless noted otherwise, and in the following, premidnight/postmidnight denote the location of a DF or S/C in the tail, while dawnside/duskside refers to the respective sides of the DFs.

For each SSO we select a 3 min interval centered at the minimum value of *B*_*z*_ (*t* = 0), which corresponds to the start time of the sharp increase in *B*_*z*_ (dipolarization). These 3 min intervals capture the main characteristics of the DFs. We perform a minimum variance analysis (MVA) [*Sonnerup and Scheible*, 1998] on the magnetic field measurements between minimum and maximum *B*_*z*_ values to obtain the normal direction **n** to the front and further select SSOs using the following criteria:

1. |*Z*_GSM_| ≤ 5*R*_*E*_ and |*Y*_GSM_| ≤ 10*R*_*E*_, since sometimes, plasma sheet-like conditions are found at high *Z* and *Y* values.
2. *V*_*x*_ ≥ 150 km/s at least for one data point and *V*_*x*_ ≥ −100 km/s over the entire 3 min interval to ensure a dominant earthward flow.
3. *λ*_int_/*λ*_min_ ≥ 4, which is the intermediate to minimum eigenvalue ratio obtained from the minimum variance analysis of the magnetic field data ±1 min around the front. We require a ratio of 4 to ensure a minimum confidence level of the estimated normal direction and, on the other hand, to have enough events to supply good statistics. *Sergeev et al.* [2006] compared the normal direction obtained from MVA, the timing method [*Harvey*, 1998], and curlometer technique [*Dunlop et al.*, 2002] and found that eigenvalue ratios greater than 4 guarantee a good agreement between them.

**Figure 1:**
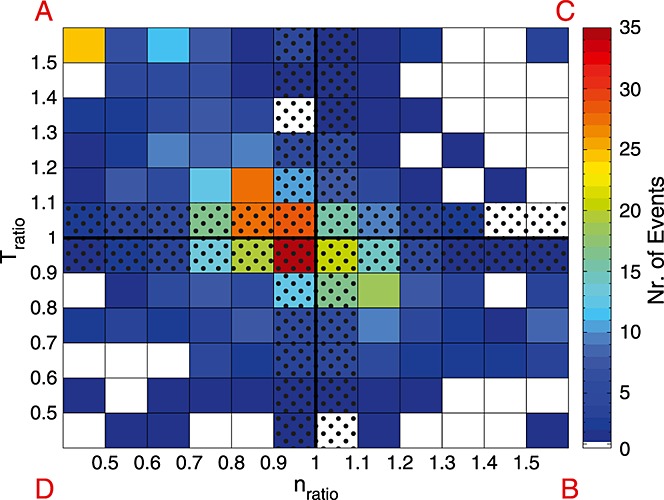
Scattered plot of the ion *T*_ratio_ and *n*_ratio_ divided into 10% bins. The dotted area mark the events which are NOT considered for the statistical analysis.

Therewith, 605 SSOs (out of 1907) remain in our data set. For every SSO we determined pre-DF and post-DF values of *T* and *n* by averaging over the first and last minutes of the respective 3 min long interval. After that, the ratios of the post-DF to pre-DF values were computed (*T*_ratio_ = *T*_post-DF_/*T*_pre-DF_ and *n*_ratio_ = *n*_post-DF_/*n*_pre-DF_). The two-dimensional histogram of the *T* and *n* ratios is shown in Figure 1.

The four quadrants in Figure 1 (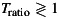 and 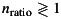), separated by the solid black lines, reflect four different categories (A–D). Category A corresponds to typical DFs [see *Runov et al.*, 2011]; however, there are also significant number where *T* and *n* change differently. While 266 SSOs (44%) fall within the categorization of *Runov et al.* [2011], where the temperature increases and the density decreases after the DF crossing, 150 SSOs (25%) fall into the complementary category B (temperature decrease with density increase after DF passage). One third of all SSOs fall into the other two categories C (60 events, 10%) and D (129 events, 21%).

SSOs outside the dotted area in Figure 1 show a clear *T* and *n* change (≥10%). These 303 SSOs (A: 166 (54%), B: 83 (28%), C: 17 (6%), and D: 37 (12%)) represent the data set for our study.

## 3. Statistical Analysis and Results

**Figure 2:**
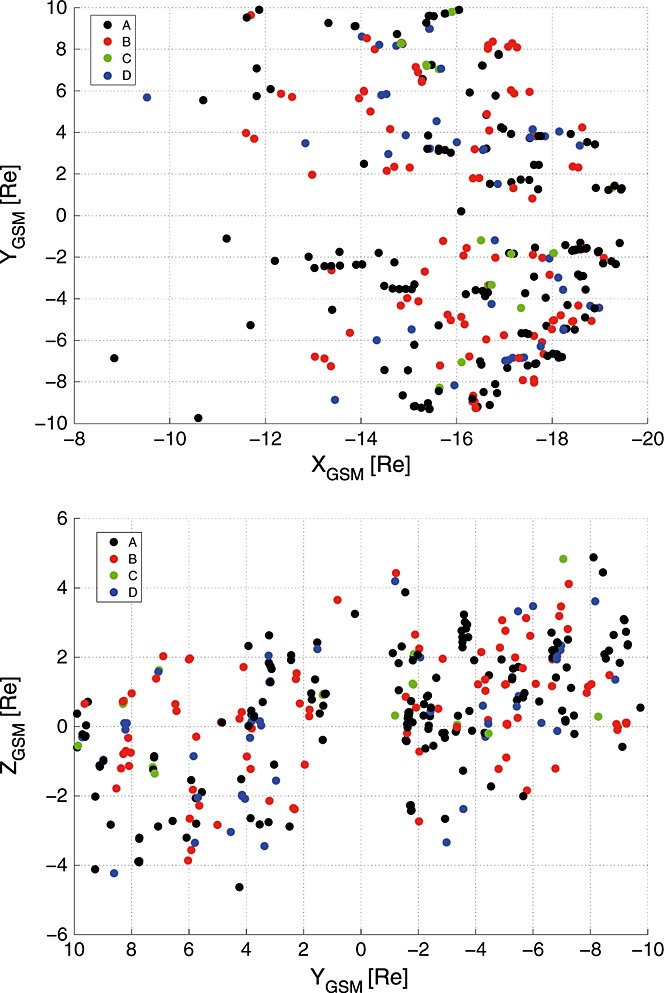
(top) *XY* and (bottom) *YZ* position of Cluster during the observations of the 303 DF events. The colors mark the four different categories with respect to the temperature and density ratio before and after the DF crossing.

The distribution of the 303 SSOs on the *XY* GSM and *YZ* GSM planes are shown in Figure 2.

As can be seen, SSOs are more abundant with increasing *X* and are observed up to a certain *XY* location. This is due to the orbit of Cluster, which crosses the plasma sheet near the apogee (∼19*R*_*E*_) between 2001 and 2006. For later years the orbit tilts southward, and therefore, Cluster crosses the plasma sheet at closer distances and spends less time in the plasma sheet. Hence, the majority of SSOs are observed at high *X* locations. Also, the asymmetry in Figure 2 (bottom), i.e., the decreasing *Z* component of SSO locations with increasing *Y*, is a result of the seasonal difference in Clusters orbit: During July/October Cluster crosses plasma sheet in the postmidnight/premidnight sector.

However, there is no clear pattern between SSOs of different categories and the location where they are observed in the tail.

**Figure 3:**
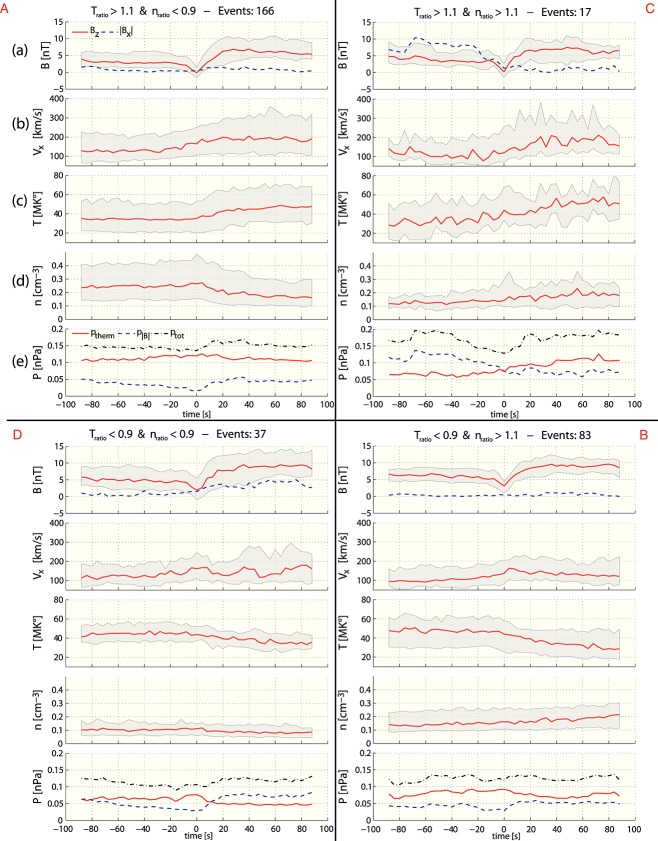
Superposed Epoch analysis of (a) the *B*_*x*_ (dashed line) and *B*_*z*_ (solid line) variations, (b) the *X* component of the ion velocity, (c) the ion temperature, and (d) the ion density. In Figure 3e the thermal (solid line) and magnetic (dashed line) pressure variations are shown. The gray lines in each panel correspond to the upper and lower quartiles. The four different categories with respect to the temperature and density ratio, are schematically illustrated by the four quadrants, divided by the solid black lines. For all categories, the same panel order as described above are applied.

Figure 3 shows a superposed epoch analysis of (a) *B*_*z*_, (b) *V*_*x*_, (c) *T*, (d) *n*, and (e) *P* over the 3 min intervals pertaining to DF SSO categories A–D. Category A reveals the same behavior as earlier studies [*Runov et al.*, 2011; *Ohtani et al.*, 2004]: The median *B*_*z*_ of the 166 SSOs shows the typical asymmetric bipolar signature with a minimum and maximum amplitude of 0 and 8nT, respectively. |*B*_*x*_| and |*B*_*y*_| (not shown) stay below 3 nT over the entire 3 min interval. The median of *V*_*x*_ increases gradually to ∼180 km/s, starting ∼60 s before the DF onset and stays approximately constant behind the front. The ion temperature and density increases and decreases rapidly across the DF in factor of ∼1.3 and ∼ 0.7, respectively. While the temperature gradually increases at the DF onset, the plasma density first increases ahead of the front and only then starts to decrease. The observed ion pressure starts to increase slightly ∼60s ahead of the front and decreases down to the initial value ∼60s after front crossing, indicating that density and temperature variations after the front crossing almost compensate. Since the temperature increases by a factor of ∼1.3 and density decreases by a factor of ∼0.7, it would be expected that the pressure remains constant per *P*_therm_ = *nk*_*B*_*T*. The median magnetic pressure increases at the DF according to the *B*_*z*_ variations and returns to almost the initial value ∼60 s after the front crossing. The total pressure, *P*_tot_ = *P*_therm_ + *P*_*B*_, minimally increases before (according to the thermal pressure) and across the DF (according to the magnetic pressure) and relaxes ∼60 s after the DF onset (*B*_*z*,min_) to almost the initial value.

The median quantities of the 83 SSOs of category B show a different behavior: *B*_*z*_ is on average ∼4nT higher than for category A, but |*B*_*x*_| and |*B*_*y*_| also stay well below 3nT over the entire 3 min interval. In contrast to category A, the maximum value of *V*_*x*_ ≈ 160 km/s is colocated with the DF onset, followed by a slight decrease over the DF. *T* and *n* decrease and increase gradually across the DF by a factor of ∼0.7 and ∼ 1.3, respectively. Interestingly, the ion density, in contrast to category A, monotonically increases over the entire 3 min interval. The ion pressure, however, shows the typical behavior: slightly increase before the front and deceases across the DF down to the initial value. The magnetic pressure, as expected, reveals the *B*_*z*_ signature and is on average higher than for category A. The total pressure remains relatively constant with slight undulations.

Note that the *V*_*x*_ profile of category A, i.e., a gradual increase over the front, and category B, i.e., a slight decrease over the front, are similar to those reported by *Fu et al.* [2012]. For category A the magnetic flux from behind the DF moves faster and accumulates at the DF, whereas for category B the peak of the BBF is colocated with the DF indicating that the magnetic flux from behind the DF moves slower and the magnetic structure gradually “dissolves.” According to the study of *Fu et al.* [2012], we interpret A as a growing dipolarizing flux bundle (DFB, region behind the DF where magnetic field strength is enhanced) and B as a decaying DFB. It should be mentioned that *Fu et al.* [2012] called this region behind the DF a flux pileup region. However, we prefer the terminology DFB from *Liu et al.* [2013b] to avoid confusion between those who claim that a flux pileup region is the region close to the Earth where the bundles/flux tubes/DFs are braking and piling up.

Now we look at the superposed epoch results of category C and D: After the front crossing, category C (category D) shows a significantly lower (higher) |*B*|, which is associated with a higher (lower) magnetic pressure, and henceforth a lower (higher) plasma *β*. For category C, Cluster moves from a small plasma beta *β* < 1 and large |*B*_*x*_| regime to a high beta *β* > 1 and low |*B*_*x*_| regime, which can be interpreted as an entry of the S/C into the central plasma sheet. Category D shows exactly the opposite behavior, and Cluster seems to leave the central plasma sheet. This means that the change in plasma parameter are not due to the DF process but due to the change in plasma region. Thus, only categories A and B are considered and investigated further.

If we assume the magnetic tension force to propel the plasma behind the DFs earthward, then the direction of that force in the GSM *XY* plane should correspond to the propagation direction of the DFs themselves. Hence, we introduce a new coordinate system, derived from GSM (rotated around *Z*_GSM_), that is aligned with the DF propagation direction. The rotation is based on the Tsyganenko 89 magnetic field model [*Tsyganenko*, 1989]. Hence, we denote this new coordinate system as T89 coordinate system {*X*_T89_,*Y*_T89_,*Z*_T89_}. In this new coordinate system, *X*_T89_ represents the direction of the magnetic tension force. *X*_T89_ is determined by the T89 magnetic field direction, which has been evaluated from the difference between the T89 magnetic field model vectors in the northern and southern lobes, ±3*R*_*E*_ above and below each SSO location (SSO *Z*_GSM_ location ±3Re), and then projected on the *XY*_GSM_ plane. The *X*_T89_ axes is chosen to be positive toward the Earth, *Z*_T89_ axes points along the *Z*_GSM_ axes, and *Y*_T89_ = *Z*_T89_ × *X*_T89_ completes the right-handed coordinate system. In this coordinate system the normal direction **n**_*x*,T89_ (from MVA) provides the front orientation relative to the moving structure. Here we chose the sign of **n**_*x*,T89_ to be positive. We denote the angle between **n**_*xy*,T89_ and *X*_T89_ as *θ*. Positive (negative) *θ* indicate that the S/C crossed the respective DF on the duskside (dawnside).

**Figure 4:**
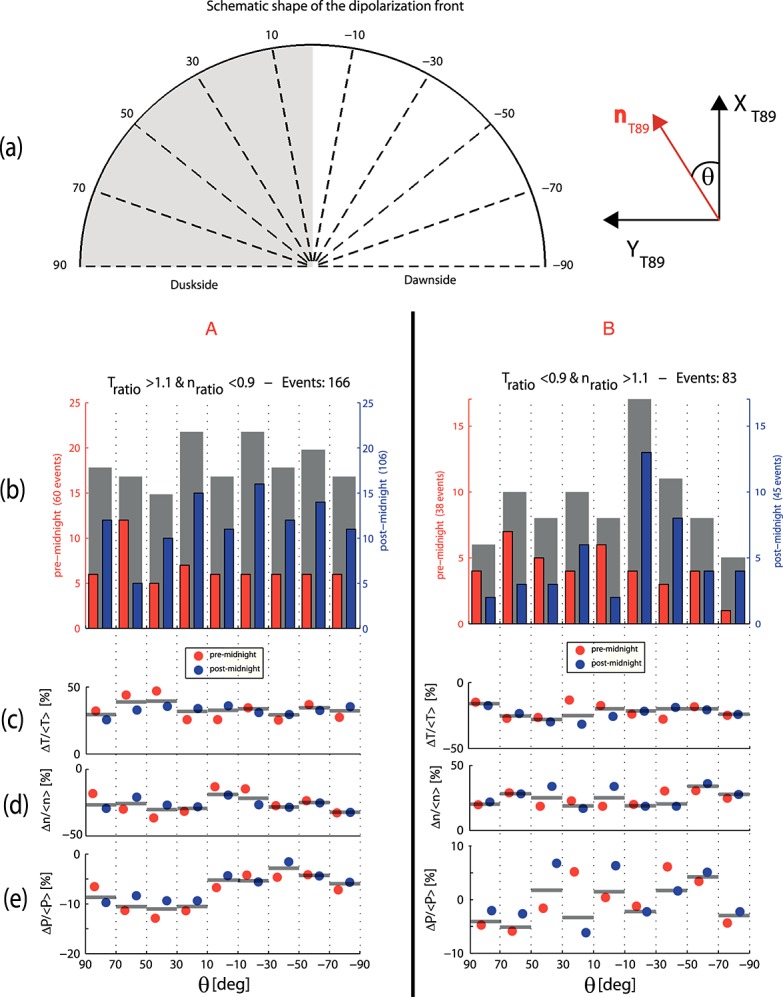
(a) Sketch of the dipolarization front with different bins used in the histograms. (b) Histograms of Cluster crossing location on the DF for category A (left) and category B (right). (c–e) The median of the relative change in ion temperature, density, and thermal pressure with respect to *θ*, respectively. *θ* denote the angle between n_*xy*,T89_ and *X*_T89_.

Figure 4a shows a sketch of the circular DF shape in *XY*_T89_ plane and the relationship of *θ*. The sectors divided by the dashed lines represent the different bins used in the histograms in Figure 4b, which represent the number of crossings with respect to *θ*. The red bars represent the SSOs observed in the premidnight sector, the blue bars events from the postmidnight sector, and the gray bars all events together. The histograms reveal that for both categories A and B, the SSOs are relatively evenly distributed with respect to *θ*. This indicates that A and B are not a result of the crossing location (i.e., A crosses only the DF duskside and B only the DF dawnside relative to the moving structure) or position in the tail (i.e., A in premidnight and B in postmidnight sector) but are, in fact, two different and independent types of DFs.

Also shown in Figure 4 is the median of the relative changes in ion temperature (Figure 4c), density (Figure 4d), and pressure (Figure 4e) during DF crossings with respect to *θ*. The relative change is defined as the difference between the median of the quantity before *B*_*z*,min_ and the median of the quantity after *B*_*z*,max_ normalized by the median of the quantity before *B*_*z*,min_.

Interestingly, the relative change in ion temperature and density is independent of *θ* and the position in the tail (premidnight or postmidnight sector) and is on average (premidnight and postmidnight sector SSOs together) 30 ± 20% and 25 ± 20% for categories A and B, respectively. All uncertainties are given by the standard deviation of the distributions.

The relative thermal pressure change, however, differs considerably between the two categories. Category A shows a clear dawn-dusk asymmetry for both premidnight and postmidnight sector observations. The maximum pressure difference is 10 ± 5% and occurs between 30° and 50° on the duskside. Toward the vertex of the DF, the pressure difference gradually decreases and stays at 5 ± 5% on the dawnside. The total pressure change (not shown), however, is independent from where the S/C crossed the DF and/or observed the DF in the tail and increases on average 10 ± 10%. Category B, on the other hand, reveals no clear pattern with respect to *θ* and/or the observation position in the tail, and the margin of error is on average ±20%. Also, the total pressure change (not shown) shows no clear pattern and increases on average 10 ± 20%. Note that the large error is caused by strong variations in the pressure between different front crossing locations. A possible explanation for these strong variations may be that category B SSOs are close to the flow-braking region where flow energy is, for example, converted to wave energy. In the case of a front with shear flow, it is well possible that this boundary is Kelvin-Helmholtz instable.

**Figure 5:**
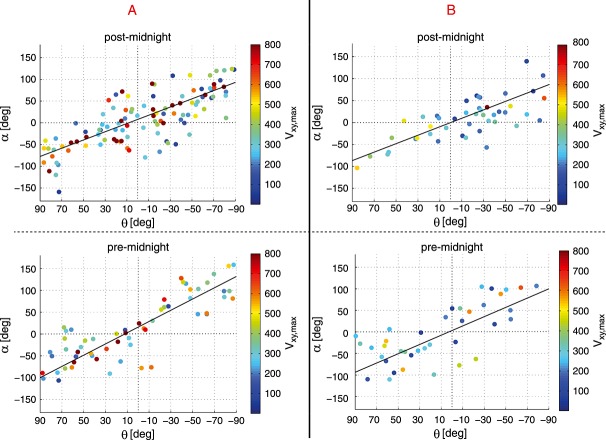
Direction and value of the maximum ion velocity in *XY* plane after *B*_*z*,min_ in the premidnight and postmidnight sector for (left column) category A and (right column) category B; *α* is the angle between the maximum plasma velocity *V*_*xy*,max_ and n_*xy*,T89_, and *θ* denotes the angle between n_*xy*,T89_ and *X*_T89_. The black line represents the least squares fit of the SSOs.

We examine the relationship between the plasma flow direction around the DF orientation. Assuming that the DF is created by the flow, we use as a representative value the maximum velocity *V*_*xy*,max_ of the flow data after *B*_*z*,min_. Figure 5 shows the angle *α* between *V*_*xy*,max_ and **n**_*xy*,T89_ (left/right columns show category A/B SSOs) as a function of *θ*. *V*_*xy*,max_ is color coded and on average (all SSOs per category) (360 ± 200)km/s and (230 ± 120)km/s for A and B, respectively. The errors are given by the standard deviation of the distributions. The black line represents the regression of the linear least squares fit of the SSOs: *α* = *k* × *θ* + *d*, where we define *k* as the convergence parameter and *d* as the flow tilt parameter. For *k* = 1 and *d* = 0 the plasma flow is everywhere around the DF parallel to the *X*_T89_ direction. For *k* < 1 the plasma flow direction diverges (plasma flows radially outward of DF center). For *k* > 1 the plasma flow after the DF converges (flow is focused toward the DF center). For *d* > 0, the flow is tilted toward the DF duskside and for *d* < 0 toward the DF dawnside. In Table 1 the fit parameters with 95% confidence bounds are given.

**Table 1:** Convergence Parameter *k* and Flow Tilt Parameter *d* (With 95% Confidence Bounds) Exhibit From the Least Squares Fit in Figure 5 Categories A and B in the Premidnight and Postmidnight Sector

		*k*	*d*
A	premidnight	1.3 ± 0.2	18 ± 8
	postmidnight	0.8 ± 0.1	8 ± 6
B	premidnight	1.1 ± 0.3	3 ± 17
	postmidnight	0.9 ± 0.2	−1 ± 10

**Figure 6:**
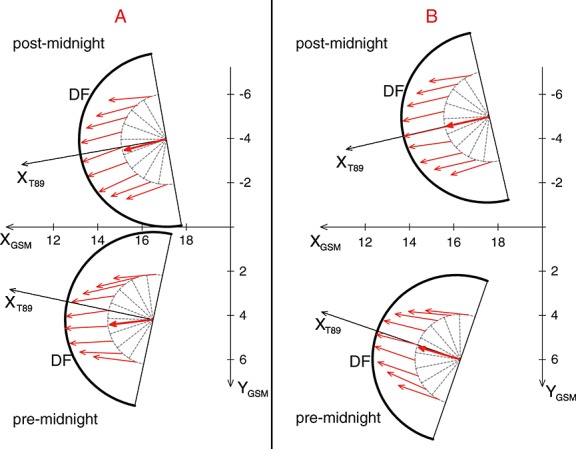
Sketch of the assumed propagation direction of the DF (*X*_T89_) and the maximum plasma flow direction in *XY* plane. The red arrows represents the direction of *V*_*xy*, max_, estimated from the least squares fit of Figure 5. The thick red arrow in the DF center is the average flow direction.

The schematic illustration of these fit parameters is depicted in Figure 6. The red arrows represent the direction of the plasma flow according to the least squares fit in Figure 5. The thick red arrow in the DF center is the average flow direction. The position and tilt of the the DF (black semicircle) is estimated from the median SSOs position and DF propagation direction (*X*_T89_).

Figure 6 comprises two main features: (1) the plasma flow direction of category A is slightly tilted toward the duskside of the DF (*d* > 0), while for category B the plasma flow is along the *X*_T89_ direction (*d* ≈ 0), and (2) in the postmidnight sector both categories show a much more divergent plasma flow (*k* ≲ 1), while in the premidnight sector the plasma flow is more convergent toward the center of the DF (*k* > 1).

Note that the error margins (with 95% confidence bounds) on the fitted parameters for the different event types overlap. This suggests that the differences in the obtained fit parameters may not be statistically significant, but they will give an indication that the flow direction of category A differs from category B. In order to evaluate the reliability of the fits, a two-tailed (lack-of-fit) *F* test at a 5% significance level has been performed [*Stigler*, 2008]. The obtained *P* values for categories A and B premidnight and postmidnight linear fits are given in Table 2. Since all *P* values are well above the 0.05 level, we can conclude that there is enough evidence that the fitting is appropriate. In addition, we determined the linear correlation coefficients (CCs) of the scattered plots. In Table 2, the absolute values of the obtained CC are given. Both statistical surveys suggest that although the errorbars are overlapping, the individual fits stand for themselves.

**Table 2:** *P* Values Obtained From the Two-Tailed (Lack-of-Fit) *F* Test at a 5% Significance Level and the Correlation Coefficients (CCs) of the Scattered Plots in Figure 5

		*P* value	CC
A	premidnight	0.42	0.87
	postmidnight	0.51	0.81
B	premidnight	0.22	0.79
	postmidnight	0.34	0.77

## 4. Discussion

**Figure 7:**
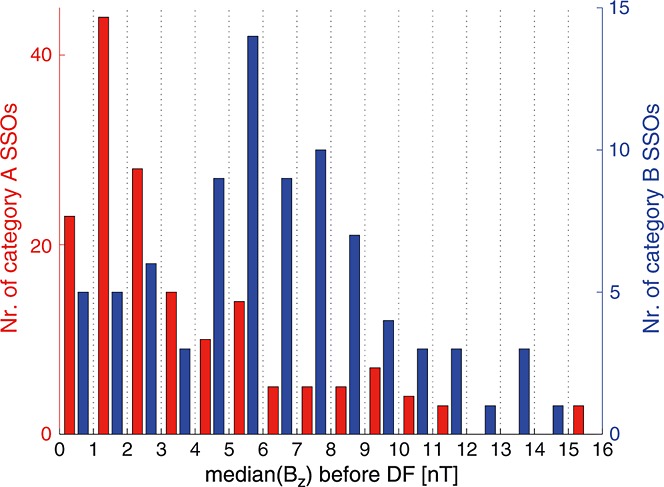
Histogram of categories A (red) and B (blue) SSOs according to their median *B*_*z*_ values before the DF crossing.

The statistical study showed that DFs are dominated by two categories. While ∼54% of the studied DFs follow the known pattern (temperature increases and density decreases across the DF), for ∼28%, the temperature decreases while the density increases across the DF. Based on the following arguments relating to these DF categories, we suggest that category A DFs may evolve into category B events and that category B is at a later stage as the DF propagates earthward from the taillike region to the near-Earth dipole region:

1. Both categories, A and B, are independent of the S/C crossing location (DF dawnside or duskside) and/or the observation position in the tail (premidnight or postmidnight). This suggests that A and B are not specific to regions in the tail, e.g., premidnight or postmidnight, but characterize individual DFs at a particular stage of evolution, regardless of the observation location.
2. Figure 3 reveals that for category A, *V*_*x*_ gradually increases over the front. That is, the magnetic flux from behind the DF moves faster and accumulates at the DF. For B, however, the maximum *V*_*x*_ is colocated with the DF onset (*t* = 0s) and decreases slightly over the DF. According to *Fu et al.* [2012], the velocity profile shown in A corresponds to the growing dipolarizing flux bundle (behind the front in the early DF stage), and the velocity profile shown in B corresponds to the decaying dipolarizing flux bundle in the later DF stage. This is supported by the observations from *Ohtani et al.* [2004], who studied fast convective flows in the plasma sheet using Geotail data between 1993 and 2001 at downtail distances between −31*R*_*E*_ < *X* < − 5*R*_*E*_. Their results indeed show that the distance between the velocity peak (maximum *V*_⊥,*x*_) and the DF decreases closer to the Earth (see their Figure 5). This suggests an evolution of the DFBs from a more growing-like type further downtail to a more decaying-like type closer to the Earth. However, in this context it is important to note that the idea of growing/decaying DFBs assumes a one-dimensional geometry of the front.
3. The maximum plasma flow velocity in *XY* plane is on average 360 ± 200km/s and 230 ± 120km/s for A and B, respectively (see Figure 5 and text above). Category A has higher velocities than category B, and we interpreted this as B occurring in a later stage after reconnection onset, since BBFs brake at near-Earth dipole field regions where background *B*_*z*_ is large.
4. Category B is in a more dipolarized region: The magnetic field before the DF is on average stronger (∼4nT) than for category A (see Figure 3). This is also confirmed by Figure 7 which shows the histogram of categories A (red) and B (blue) SSOs according to their median *B*_*z*_ values before the DF crossing. The peaks of the SSO distribution for categories A (red) and B (blue) differ by ∼4nT.
5. The thermal pressure perturbations for categories A and B (see Figure 4) also supports our hypothesis: Category A shows a similar dawn-dusk asymmetry as *Zhou et al.* [2014], who explained the asymmetry in the context of ion acceleration and reflection at DFs. Their model suggests that ions in the DF duskside pass a longer way along the motional electric field, which is carried by the DF *E*_*y*_ = *V*_*x*_*B*_*z*_ [see also *Runov et al.*, 2009]. Hence, the ions get accelerated significantly more in the DF's duskside, which results in dawn-dusk asymmetry and an enhanced thermal pressure at the duskside in front of the DF. In our study we have estimated the relative thermal pressure change over the DF crossing 
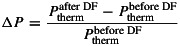
A negative Δ*P* indicates that the pressure before the DF crossing is higher than after the DF crossing. Indeed, we have found that for category A the thermal pressure is over the entire DF structure (DF dawnside and duskside) higher before the DF crossing (see Figure 4) with a higher thermal pressure at the DF duskside. This can be interpreted as the high plasma velocity of category A SSOs yields a larger electric field, and thus, a clearer dawn-dusk pressure asymmetry emerges. On the contrary, for category B SSOs, the plasma velocity is smaller and thus the electric field. In this context, it should be noted that despite the plasma velocity, the magnetic field determines the convection electric field. Although *V*_*x*_ of category A is higher compared to category B, *B*_*z*_ is smaller (see Figure 3). Nonetheless, the convection electric field, which is estimated from the median *V*_*x*_ and *B*_*z*_ values after *B*_*z*,max_, is on average ∼ 20% smaller for category B. Hence, the ions acquire less energy and the asymmetry is not that much pronounced, which may explain why category B does not show this asymmetry.
  It is also apparent in Figure 4 that the thermal pressure change of category B varies strongly between different crossing locations, indicating a disordered structure, which might be expected closer to the flow-braking region. As the flow starts to brake, this might suggest that the DF structure becomes unstable, e.g., due to the conversion of the flow energy to wave energy and thus asymmetry in the thermal pressure around the DF gets lost.
6. Our observations suggest that the plasma flow direction of category B is along the magnetic tension force direction, *X*_T89_, while for category A the plasma flow is slightly tilted toward the DF duskside (see Figure 6). Duskward deflection of the reconnection jets have been observed for the first time in hybrid simulations by *Nakamura et al.* [1998]. Also, recent particle simulations [*Sitnov et al.*, 2013; *Drake et al.*, 2014] show a duskward deflected flow. A possible explanation for the flow tilt may be the magnetic curvature drift, which turns the jet duskward [see *Drake et al.*, 2014, and references therein]. The flow tilt and magnitude will probably depend on the width of the initial current sheet: A thinner current layer will lead to smaller curvature radius and thus to a larger curvature force. On the other hand, a thicker current layer will yield a smaller curvature force and the duskward flow tilt disappears. Accordingly, we interpret that category A is closer to the reconnection region where the current sheet is thinner, while category B is closer to the flow-braking region with a broader current layer.

Based on these results, we hypothesize that category A evolves into category B. Simplified, this can be described on the basis of Figure 1: The *T* and *n* ratios change from those described in the top left corner to those at the center, as SSOs propagate toward Earth.

**Figure 8:**
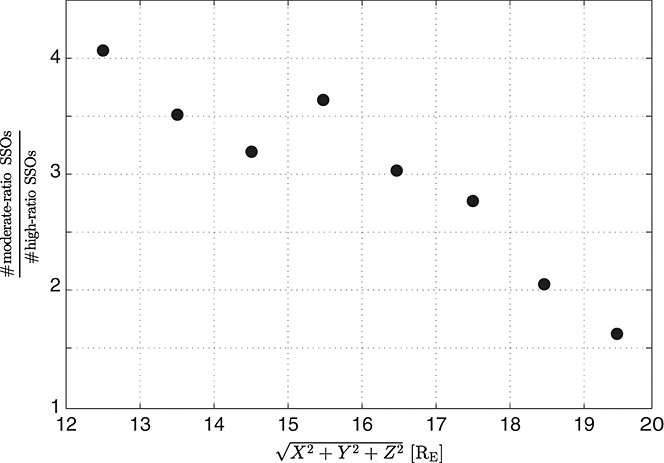
Binned ratio of the numbers of “moderate-ratio” SSOs (0.8 < *T*_ratio_ < 1.2; 0.8 < *n*_ratio_ < 1.2) to the number of “high-ratio” SSOs (*T*_ratio_ ≥ 1.2; *n*_ratio_ ≤ 0.8) with a bin size of 1*R*_*E*_.

However, some observations seem to contradict this hypothesis: Although category B is expected to be closer to the Earth (since they are at a later stage), some of category A SSOs are closer (see Figure 2). One explanation might be that these SSOs stay longer in “category A stage.” Indeed, a statistical analysis of the radial distribution of category A SSOs reveals that “high-ratio” SSOs (*T*_ratio_ ≥ 1.2; *n*_ratio_ ≤ 0.8) are farther away from Earth than SSOs with a moderate ratio (0.8 < *T*_ratio_ < 1.2; 0.8 < *n*_ratio_ < 1.2) (see Figure 8). Furthermore, the spatial configuration and position of the near-Earth tail magnetic field varies significantly, altering the transition region from stretched to dipolar field lines [see, e.g., *Takada et al.*, 2006] and therefore the location of the flow-braking region. Thus, the simple statistical study of radial distribution of the SSOs in the tail cannot describe the pattern of the radial evolution of the flow on a case by case basis.

Therefore, we assume an evolutionary process for category A with high *T* and *n* ratios to moderate *T* and *n* ratios, becoming category B SSOs. Most of the SSOs are either high *T* and *n* ratio SSOs from category A (top left corner in Figure 1) or moderate SSOs from categories A or B (center in Figure 1). We interpret this as follows: High *T* and *n* ratio SSOs from category A are observed just after reconnection, where the tenuous plasma from the lobe gets accelerated and heated. For moderate SSOs, however, the plasma conditions before and after the front are almost in equilibrium and the plasma flow is about to brake (decaying dipolarizing flux bundle).

## 5. Conclusion

From the results obtained in this study, we suggest that DFs with high ratios of the plasma *T* and *n* from before to after the front (category A) evolve into low *T* and *n* ratio DFs (typical description for category B), as the flow/DF propagates earthward. We thus hypothesize that after (transient) reconnection takes place, a BBF emerges with category A characteristics, and as it travels earthward, it evolves to have category B characteristics, which is in a more dipolarized region (closer to the flow-braking region).

The idea of such a DF evolution process, however, needs to be investigated in detail in a further work by multipoint measurements with greater separations along the *X* direction (e.g., THEMIS). This topic should be also addressed by future multispacecraft magnetospheric missions, such as MMS, to investigate the reconnection process in detail and to reveal how DFs are formed.

## Key Points

- DFs fall mainly in two categories with respect to *T* and *n* variations across the DF
- Categories are independent of S/C crossing location and/or position in the tail
- Idea: Category with orginal BBF source characteristic evolves into other category

## Appendix

### Appendix A: ECLAT In Situ Data Products: Cluster Event Identification

The European Cluster Assimilation Technology (ECLAT) project is funded by the Seventh Framework Programme of the European Union to provide contextual data sets for inclusion in the Cluster Active Archive (CAA). Amongst others, the ECLAT project offers in situ Cluster data products in the form of magnetotail plasma region identifications: data plasma regions and event lists. The event catalogues have been delivered to various public access ECLAT data portals and will be incorporated into the CAA by the European Space Agency.

In the following, we will give a brief summary of some significant selection criteria used for the DF identification. For additional information, please see the report “ECLAT Cluster event identification report (D210.1)” on the website http://www.eclat-project.eu or contact the corresponding author (daniel.schmid@oeaw.ac.at).

The starting data sets for the event search are the CAA Cluster 4 s averaged magnetic field data obtained by FGM and plasma data from Cluster Ion Spectrometry (CIS)-HIA when Cluster is in the tail (July–October) for 2001–2009. Clusters 1 and 3 only are used to identify DFs (reference spacecraft) due to the poorer resolution and quality of plasma data on other spacecraft. To find the DF events, similar selection criteria as in *Schmid et al.* [2011] are used. At the beginning, a 3 min long sliding window with an increment of 30 s has been used, where the following requirements on the FGM data set had to be accomplished:

1. the spacecraft is located between −19*R*_*E*_ ≤ *X*_GSM_ ≤ −8*R*_*E*_ and |*Y*_GSM_| ≤ 15*R*_*E*_,
2. the difference in elevation angle (Θ = tan^−1^(*B*_*z*_/*B*_*x**y*_)) between minimum and maximum *B*_*z*_ during the window exceeds ΔΘ ≥ 10° and Δ*B*_*z*_ also exceeds 4nT,
3. the time at the maximum *B*_*z*_ is greater than the time at the minimum *B*_*z*_,
4. the elevation angle is at least in one data point (out of 45 data points in the 3 min window) greater than Θ ≥ 45°, and
5. data gaps within the window are not allowed.

Tail plasma region files resulting from WP220 “In Situ data products: Cluster region and boundary identification” of the ECLAT project have been used to maintain that the reference spacecraft remains in the plasma sheet (outer or inner) [*Boakes et al.*, 2014]. Subsequently, the following requirements on the CIS-HIA plasma data set of the reference spacecraft had to be satisfied: (1) the absolute value of the *x* component of the plasma flow velocity |*V*_*x*_| is at least in one data point (out of 45 data points in the 3 min window) greater than |*V*_*x*_| ≥ 100 km/s and (2) data gaps within the window are not allowed.

This selection criterion therefore limits DFs with a timescale of *B*_*z*_ enhancement (monotonic increase) up to 3 min scale. For each event found by the reference spacecraft, Cluster 1 or 3, the magnetic field conditions above only (no plasma condition) are used to check if the same event can be found in each other spacecraft within the event interval of the reference spacecraft.

## References

[b1] Angelopoulos V, Baumjohann W, Kennel C F, Coroniti F V, Kivelson M G, Pellat R, Walker R J, Lühr H, Paschmann G (1992). Bursty bulk flows in the inner central plasma sheet. J. Geophys. Res.

[b2] Balogh A (2001). The Cluster magnetic field investigation: Overview of in-flight performance and initial results. Ann. Geophys.

[b3] Baumjohann W (1993). The near-Earth plasma sheet: An AMPTE/IRM perspective. Space Sci. Rev.

[b4] Baumjohann W (2002). Modes of convection in the magnetotail. Phys. Plasmas.

[b5] Baumjohann W, Hesse M, Kokubun S, Mukai T, Nagai T, Petrukovich A A (1999). Substorm dipolarization and recovery. J. Geophys. Res.

[b6] Baumjohann W, Schödel R, Nakamura R (2002). Bursts of fast magnetotail flux transport. Adv. Space Res.

[b7] Boakes P D, Nakamura R, Volwerk M, Milan S E (2014). ECLAT Cluster Spacecraft Magnetotail Plasma Region Identifications (2001–2009). Dataset Pap. Sci., 2014.

[b8] Drake J F, Swisdak M, Cassak P A, Phan T D (2014). On the 3-D structure and dissipation of reconnection-driven flow bursts. Geophys. Res. Lett.

[b9] Dunlop M W, Balogh A, Glassmeier K-H, Robert P (2002). Four-point cluster application of magnetic field analysis tools: The Curlometer. J. Geophys. Res.

[b10] Fu H S, Khotyaintsev Y V, Vaivads A, André M, Sergeev V A, Huang S Y, Kronberg E A, Daly P W (2012). Pitch angle distribution of suprathermal electrons behind dipolarization fronts: A statistical overview. J. Geophys. Res.

[b11] Harvey C C, Paschmann G, Daly P (1998). Spatial gradients and the volumetric tensor. Analysis Methods for Multi-Spacecraft Data.

[b12] Kim H-S, Lee D-Y, Ohtani S-I, Lee E-S, Ahn B-H (2010). Some statistical properties of flow bursts in the magnetotail. J. Geophys. Res.

[b13] Li S-S, Angelopoulos V, Runov A, Zhou X-Z, McFadden J, Larson D, Bonnell J, Auster U (2011). On the force balance around dipolarization fronts within bursty bulk flows. J. Geophys. Res.

[b14] Liu J, Angelopoulos V, Runov A, Zhou X-Z (2013a). On the current sheets surrounding dipolarizing flux bundles in the magnetotail: The case for wedgelets. J. Geophys. Res. Space Physics.

[b15] Liu J, Angelopoulos V, Zhou X Z, Runov A, Yao Z (2013b). On the role of pressure and flow perturbations around dipolarizing flux bundles. J. Geophys. Res. Space Physics.

[b16] Nakamura M S, Fujimoto M, Maezawa K (1998). Ion dynamics and resultant velocity space distributions in the course of magnetotail reconnection. J. Geophys. Res.

[b17] Nakamura R (2002). Motion of the dipolarization front during a flow burst event observed by cluster. Geophys. Res. Lett.

[b18] Nakamura R, Rentinó A, Baumjohann W, Volwerk M, Erkaev B K N, Lucek E A, Dandourasn I, André M, Khotyaintsev Y (2009). Evolution of dipolarization in the near-Earth current sheet induced by Earthward rapid flux transport. Ann. Geophys.

[b19] Ohtani S, Shay M A, Mukai T (2004). Temporal structure of the fast convective flow in the plasma sheet: Comparison between observations and two-fluid simulations. J. Geophys. Res.

[b20] Pontius D H, Wolf R A (1990). Transient flux tubes in the terrestrial magnetosphere. Geophys. Res. Lett.

[b21] Rème H (2001). First multispacecraft ion measurements in and near the Earth's magnetosphere with the identical Cluster Ion Spectrometry (CIS) experiment. Ann. Geophys.

[b22] Runov A, Angelopoulos V, Sitnov M I, Sergeev V A, Bonnell J, McFadden J P, Larson D, Glassmeier K-H, Auster U (2009). THEMIS observations of an earthward-propagating dipolarization front. Geophys. Res. Lett.

[b23] Runov A, Angelopoulos V, Zhou X Z, Zhang X J, Li S, Plaschke F, Bonnell J (2011). A THEMIS multicase study of dipolarization fronts in the magnetotail plasma sheet. J. Geophys. Res.

[b24] Schmid D, Volwerk M, Nakamura R, Baumjohann W, Heyn M (2011). A statistical and event study of magnetotail dipolarization fronts. Ann. Geophys.

[b25] Sergeev V A, Sormakov D A, Apatenkov S V, Baumjohann W, Nakamura R, Runov A, Mukai T, Nagai T (2006). Survey of large-amplitude flapping motions in the midtail current sheet. Ann. Geophys.

[b26] Sergeev V, Angelopoulos A V, Nakamura R (2012a). Recent advances in understanding substorm dynamics. Geophys. Res. Lett.

[b27] Sergeev V A, Chernyaev I A, Dubyagin S V, Miyashita Y, Angelopoulos V, Boakes P D, Nakamura R, Henderson M G (2012b). Energetic particle injections to geostationary orbit: Relationship to flow bursts and magnetospheric state. J. Geophys. Res.

[b28] Sigsbee K, Slavin J A, Lepping R P, Szabo A, Øieroset M, Kaiser M L, Reiner M J, Singer H J (2005). Statistical and superposed epoch study of dipolarization events using data from Wind perigee passes. Ann. Geophys.

[b29] Sitnov M I, Buzulukova N, Swisdak M, Merkin V G, Moore T E (2013). Spontaneous formation of dipolarization fronts and reconnection onset in the magnetotail. Geophys. Res. Lett.

[b30] Sonnerup B U Ö, Paschmann G, Daly P, Scheible M (1998). Minimum and maximum variance analysis. Analysis Methods for Multi-Spacecraft Data.

[b31] Stigler S (2008). Fisher and the 5% level. CHANCE.

[b32] Takada T, Nakamura R, Baumjohann W, Asano Y, Volwerk M, Zhang T L, Klecker B, Rème H, Lucek E A, Carr C (2006). Do BBFs contribute to inner magnetosphere dipolarizations: Concurrent cluster and double star observations. Geophys. Res. Lett.

[b33] Tsyganenko N A (1989). A magnetospheric magnetic field model with a warped tail current sheet. Planet. Space Sci.

[b34] Volwerk M (2008). Magnetotail dipolarization and associated current systems observed by cluster and double star. J. Geophys. Res.

[b35] Zhou X-Z, Angelopoulos V, Liu J, Runov A, Li S S (2014). On the origin of pressure and magnetic perturbations ahead of dipolarization fronts. J. Geophys. Res. Space Physics.

